# Metabolomic evaluation of different starter culture effects on water-soluble and volatile compound profiles in *nozawana* pickle fermentation

**DOI:** 10.1016/j.fochms.2021.100019

**Published:** 2021-03-17

**Authors:** Satoru Tomita, Jun Watanabe, Takeshi Kuribayashi, Sachi Tanaka, Takeshi Kawahara

**Affiliations:** a Food Research Institute, National Agriculture and Food Research Organization (NARO), 2-1-12 Kannondai, Tsukuba, Ibaraki 305-8642, Japan; b Food Technology Department, Nagano Prefecture General Industrial Technology Center, 205-1 Kurita, Nagano, Nagano 380-0921, Japan; c Faculty of Agriculture, Shinshu University, 8304 Minamiminowa, Kamiina, Nagano 399-4598, Japan; d Academic Assembly School of Science and Technology, Institute of Agriculture, Shinshu University, 8304 Minamiminowa, Kamiina, Nagano 399-4598, Japan

**Keywords:** Fermented food, Fermented pickle, Cruciferous vegetables, Lactic acid bacteria, Metabolomics, NMR, SPME-GC/MS, Isothiocyanates

## Abstract

Metabolomic characterization of a lactic-fermented pickle of *nozawana* (*Brassica rapa* L. var. *hakabura*) was conducted to evaluate the effects of different starter culture strains on the chemical profiles. We compared the profiles of water-soluble and volatile compounds obtained by non-targeted nuclear magnetic resonance and solid-phase microextraction gas chromatography/mass spectrometry analyses. Principal component analyses indicated that the fermented samples differed significantly in terms of the levels of various compounds, including taste- and aroma-active components, such as water-soluble residual sugars, organic acids, mannitol, ethanol, dihydroxyacetone, ornithine, γ-aminobutyric acid, choline, volatile isothiocyanates, 3,4-epithiobutyl cyanide, 2,3-butanedione, acetoin, ethyl acetate, dimethyl trisulfide, and *S*-methyl thioacetate. Fermentation with a *Latilactobacillus curvatus* culture was associated with a unique metabolite profile characterized by higher levels of isothiocyanates and hexanoic acid and lower levels of lactic acid, acetic acid, acetoin, and 2,3-butanedione. These variations in the chemical profile might be associated with different qualities in fermented *nozawana* pickle products.

## Highlights

• Fermented *nozawana* pickle with starter culture strains was studied by metabolomics.
• NMR and SPME-GC/MS analyses revealed starter culture-dependent metabolite profiles.
• Various flavor compounds showed significantly different levels among the strains.
• *Latilactobacillus curvatus* exhibited higher isothiocyanate intensities at a maximum 3.30-fold.
• *Levilactobacillus brevis* influenced on dimethyl trisulfide and *S*-methyl thioacetate levels.

## 1 Introduction

*Nozawana* (*Brassica rapa* L. var. *hakabura*) is a variety in the cruciferous plant family that is common in foods worldwide and is an economically and culturally important vegetable. Pickled vegetables are popular processed food products in Japan and elsewhere, and pickled *nozawana* (called *nozawana-zuke*) enjoys the largest market share in Japan among pickled leafy vegetables. A traditional method for preparing *nozawana-zuke* is fermentation with lactic acid bacteria (LAB). Currently, however, most *nozawana-zuke* products are manufactured without fermentation as quick-pickled *nozawana-zuke*.

Nonetheless, fermented *nozawana-zuke* remains popular because of its complex flavor profile that develops during lactic acid fermentation and that is unique to each producer. In practical manufacturing, fermented *nozawana-zuke* is prepared by spontaneous fermentation utilizing the activity of autochthonous LAB rather than a starter culture, likely derived from raw materials. Although conventional spontaneous fermentation is a simple and low-cost alternative to the starter culture method, it involves management of the fermentation process and product quality due to uncontrollable bacterial communities. To stabilize the fermentation process and product quality of fermented *nozawana-zuke*, it is essential to understand the bacterial community structure and the impact of various LAB on the chemical composition.

Several studies have analyzed the bacterial communities present in spontaneously fermented *nozawana-zuke*. Recently, a culture-independent analysis of LAB in fermented *nozawana-zuke* found that *Latilactobacillus curvatus* (formerly *Lactobacillus curvatus*) was dominant, followed by *Lactiplantibacillus plantarum* (formerly *Lactobacillus plantarum*) and *Levilactobacillus brevis* (formerly *Lactobacillus brevis*), and occasionally by *Latilactobacillus sakei* (formerly *Lactobacillus sakei*) (Sandagdorj, Hamajima, Kawahara, Watanabe, & Tanaka, 2019). A study employing a culture-dependent method isolated *Leuconostoc mesenteroides* and *Latilactobacillus curvatus* as dominant LAB and detected no LAB of the genera *Streptococcus*, *Pediococcus*, or *Lactococcus* (Osawa, Takanami, Kuribayashi, & Kuwahara, 2005). A bacteriocin-producing LAB strain isolated from fermented *nozawana-zuke* has also been identified as *Latilactobacillus curvatus* (Kawahara, Iida, Toyama, & Fukuda, 2010). In an earlier study, *Leuconostoc mesenteroides* and *Limosilactobacillus fermentum* (formerly *Lactobacillus fermentum*) were isolated from commercial *nozawana-zuke* products (Nakagawa et al., 2001). The results of these studies also indicated that the bacterial composition of spontaneously fermented *nozawana-zuke* changed depending on the duration of fermentation. In the fermentation of sauerkraut, which is one of the most studied fermented pickles of cruciferous vegetable, it is well known that the bacterial composition, in general, shows a successive growth of several different LAB species including *Leuconostoc mesenteroides*, *Levilactobacillus brevis*, and *Lactiplantibacillus plantarum* (Pederson & Albury, 1969). Additionally, the chemical characteristics (total acid production and the ratio of organic acids) are associated with the succession and the changes in composition of LAB species resulting from different fermentation conditions such as temperature and NaCl concentration (Pederson & Albury, 1969).

These microbiological studies on *nozawana-zuke* suggest that these LAB are important facilitators of *nozawana-zuke* fermentation and can be potentially used as starter cultures to control chemical composition. However, to the best of our knowledge, the impact of these LAB on the compositional characteristics of *nozawana-zuke* has not yet been empirically assessed, and the components that exhibit common or different changes among the starter culture strains remain unclear. Considering the biodiversity of LAB (Claesson et al., 2007, Gänzle, 2009), it is hypothesized that LAB play multiple roles in *nozawana-zuke* fermentation, leading to differences in the metabolite profile. In recent years, comprehensive compositional analysis via the metabolomics approach has been performed in studies on various fermented foods, including fermented pickles (Mozzi et al., 2013, Park et al., 2016, Randazzo et al., 2017). The application of metabolomic analysis to clarify the changes in a wide range of metabolites produced through starter culture fermentation of *nozawana-zuke* is promising.

*Nozawana* is a popular, locally cultivated ingredient long since established as a traditional regional vegetable in the Nagano Prefecture of Japan. This study analyzed and compared the compositional profiles of *nozawana-zuke* samples fermented by four different starter cultures alongside a sample fermented by a non-starter culture to assess the potential impacts of starter culture LAB on the chemical characteristics. Compositional analysis was conducted using non-targeted metabolomics based on a combined application of nuclear magnetic resonance (NMR) spectroscopy and solid-phase microextraction (SPME) gas chromatography/mass spectrometry (GC/MS) to obtain in-depth compositional profiles of water-soluble and volatile components.

## 2 Materials and methods

### 2.1 Bacterial strains

The LAB strains used in this study were isolated from homemade and commercially available *nozawana-zuke* produced in Nagano, Japan, as described previously (Sandagdorj, et al., 2019). The four isolates used for starter cultures were *Lactiplantibacillus plantarum* K4G4 and K5G3, *Latilactobacillus curvatus* ♯4G2, and *Levilactobacillus brevis* K4G1. The taxonomy of the four strains was verified by 16S rRNA gene sequencing and species-specific polymerase chain reaction assays (Berthier and Ehrlich, 1999, Fusco et al., 2016, Torriani et al., 2001). Cultures were grown in Lactobacilli MRS Broth (Difco Laboratories, Detroit, MI, USA). Plate Count Agar with BCP (Nissui Pharmaceutical Co., Tokyo, Japan) was used to measure viable LAB cell counts of the fermented samples.

### 2.2 Fermentation of *nozawana-zuke*

*Nozawana-zuke* fermentation was performed based on a two-step, well-established industrial procedure for pre-salting and fermentation. Fresh *nozawana* raw material was obtained from Takeuchi Nosan Corp. (Nagano, Japan), washed with water, and chopped into 2.5-cm sections. Sections were washed in 100 ppm NaClO (Sankyo Co., Nagano, Japan) for 3 min, thoroughly rinsed with water, and then salted with 10 kg of 6% brine per 20 kg of material for 2 d at 7 °C. The material was again rinsed with water, and the viable cells in the pre-salted material were confirmed to be <1 × 10^3^ CFU/g. Then, portions of 200 g of material and 100 g of brine were placed in heat-sealed nylon bags (BA-1727H; Meiwa Pax Co. Ltd., Osaka, Japan), the salt concentration was adjusted to 1.5%, and starter cultures was added at a population of 1 × 10^5^ CFU/g. Separate bags containing only the material and brine were used as controls. Three bags were prepared for each of the four bacterial strains and non-starter control, and the samples were fermented at 10 °C for 7, 14, or 21 d. Separate bags were used for each fermentation period in single replicate to avoid potential contamination from iterative handling.

After fermentation, the bag components were gently homogenized in a stomacher (Masticator 400S; Gunze Sangyo, Inc., Tokyo, Japan) for 1 min at room temperature, and the liquid was decanted from each bag. The liquid samples were analyzed to measure pH, titratable acidity (TA) calculated as lactic acid, and viable LAB cell counts. The samples were then subjected to metabolomic characterization.

### 2.3 NMR analysis

Water-soluble compounds were analyzed by NMR spectroscopy as described previously but with minor modifications (Tomita et al., 2015). Briefly, NMR spectra were measured on an Avance III 500 MHz (Bruker, Billerica, MA, USA) equipped with a CryoProbe CPBBO and SampleJet automatic sample changer (Bruker). Analytical sample preparation was performed using a previous method with minor modifications (Tomita, Nakamura, & Okada, 2018). Briefly, 130 µL of sample supernatant centrifuged at 20,500×*g* for 5 min at 4 °C was mixed with 520 µL of 125 mM potassium phosphate buffer (pH 7.0) in deuterium oxide (99.9% D; Cambridge Isotope Laboratories, Andover MA, USA). After centrifuging again, 600 µL of clear supernatant was transferred to NMR sample tubes (5.0 mm O.D. × 103.5 mm; Norell, Landisville, NJ, USA). Proton (^1^H) NMR spectra were recorded using a Bruker pulse program zgpr with the following parameters: spectral width, 20 ppm; offset frequency, 4.7 ppm; ^1^H 90° pulse, 13.5 µs; relaxation delay, 4 s; and number of scans, 128. Metabolite annotation (tentative identification) was performed as described previously (Tomita, et al., 2015). Two-dimensional ^1^H–^13^C heteronuclear single quantum coherence spectra were also recorded and used for metabolite annotation as described previously (Tomita et al., 2018, Tomita et al., 2015).

### 2.4 SPME-GC/MS analysis

Profiles of volatile compounds were obtained using a previously described method with minor modifications (Tomita, et al., 2018). Briefly, 3 mL of sample was transferred to a 20-mL screw cap vial (Shimadzu GLC Ltd., Tokyo, Japan) and stored at 4 °C until use. Then, samples were placed in an agitator unit at 50 °C for 10 min, and volatile components were extracted for 20 min onto a 2-cm long DVB/CAR/PDMS fiber (Sigma-Aldrich, St. Louis, MO, USA). The agitator unit was rotated at 250 rpm during extraction. Volatile compounds were desorbed from the fiber for 3 min at 230 °C in splitless mode and were resolved on an Rtx-WAX capillary column (60 m × 0.25 mm I.D. × 0.25 μm film thickness; Restek, Bellefonte, PA, USA) with helium as the carrier gas. The column temperature program was as follows: 40 °C for 5 min, then increased to 230 °C at 5 °C/min, and maintained at 230 °C for 5 min. Analyses were conducted in duplicate to confirm reproducibility. Detected peaks were annotated based on the mass spectrum similarity and retention index in the NIST 02 MS Library (National Institute of Standards and Technology, Gaithersburg, MD, USA).

#### 2.4.1 Dataset preparation and multivariate analysis

Datasets for multivariate analyses were prepared from ^1^H NMR spectra and GC/MS chromatograms. For water-soluble compounds, NMR spectra were subdivided into 0.04 ppm integral regions (buckets), and signal intensities in each bucket were calculated using Amix Software (Bruker). Buckets were normalized against the signal intensity of the internal standard DSS-d_6_ at 0.00 ppm, and buckets with residual solvent signals were replaced with zeroes. Buckets generated from the spectral noise region with a maximum signal intensity of <0.005 were removed to eliminate noise contamination. For volatile compounds, baseline correction and peak alignment of GC/MS chromatograms were performed using MetAlign (Lommen, 2009). Mass peaks derived from a single compound were integrated using AIoutput (Tsugawa, Bamba, Shinohara, Nishiumi, Yoshida, & Fukusaki, 2011) at a height threshold of 5000 and an RSD filter value of 100. After these processes, the NMR and GC/MS datasets comprised 119 buckets and 127 peaks, respectively. Principal component analysis (PCA) was performed using SIMCA software (version 14; Umetrics, Umeå, Sweden) with Pareto scaling.

### 2.5 Statistical analysis

Integral values from highlighted metabolites were obtained by manually subdividing spectra using the variable-sized buckets option in Amix software (Bruker) by specifying spectral ranges containing an isolated signal of a single compound. For GC/MS analysis, chromatograms were processed using GCMS solution software (Shimadzu Co., Kyoto, Japan), and the peak areas of volatile compounds were recorded for signal intensity values. Statistical significance was analyzed using Tukey’s multiple comparison test with R software (version 3.6.1; R Core Development Team, Vienna, Austria), and a 0.05 level of probability was used as the criterion for significance.

## 3 Results

### 3.1 Fermented *nozawana-zuke* preparation

**Fig. 1:**
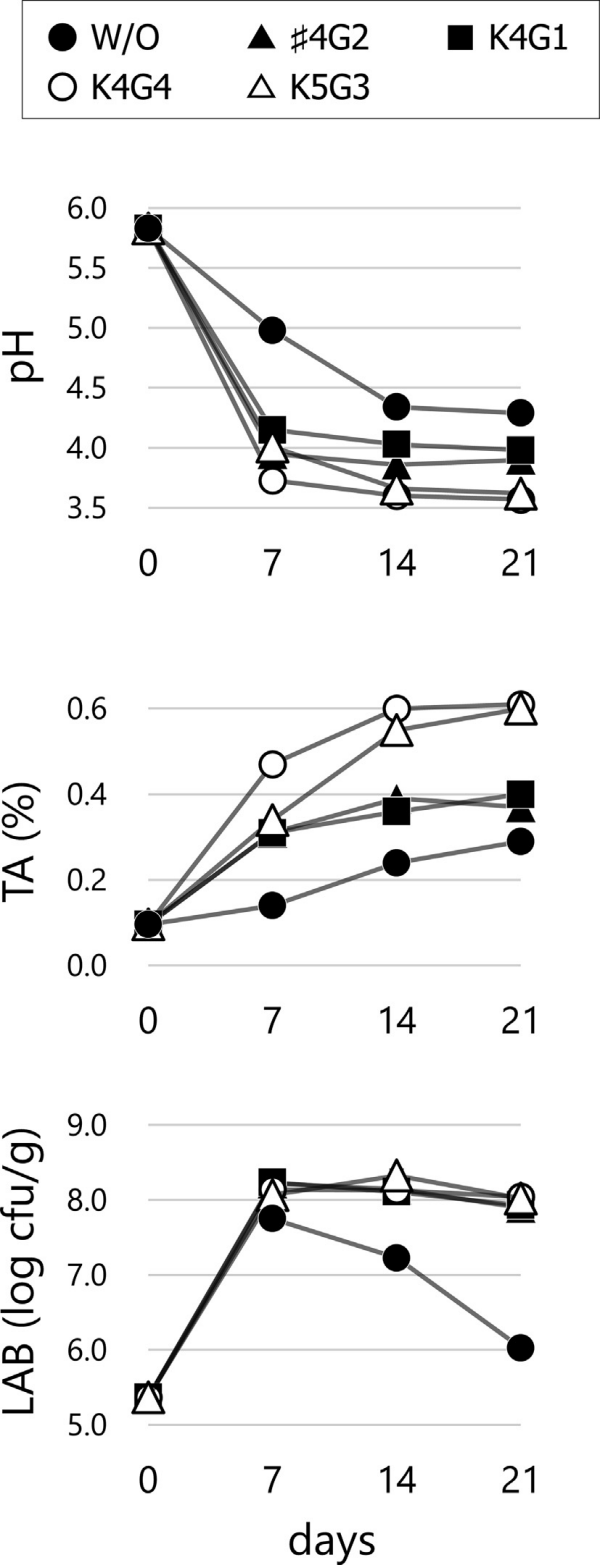
Changes in pH, titratable acidity, and viable cell counts of *nozawana-zuke* fermented with or without starter cultures. The result of viable LAB cell counts is depicted as average value of two analytical replicates.

Samples were collected from the pickling liquid of five *nozawana-zuke* fermentations with one of four starter cultures or without a starter culture (control) at different time points during fermentation. The decreases in pH and increases in TA varied among the samples (Fig. 1). The pH of starter culture samples (S/C) rapidly decreased over the first 7 d to approximately 4.0. The sample without a starter culture (W/O) presented with less prominent TA increases over a greater period of time, and the pH did not decrease to the same level as that in the S/C samples. Viable LAB cell counts were comparable among all samples for the first 7 d. However, LAB cell counts were maintained only in the S/C samples throughout the experimental period, and the W/O sample presented with decreased cell counts after 7 d.

### 3.2 Compositional analysis of fermented *nozawana-zuke*

**Fig. 2:**
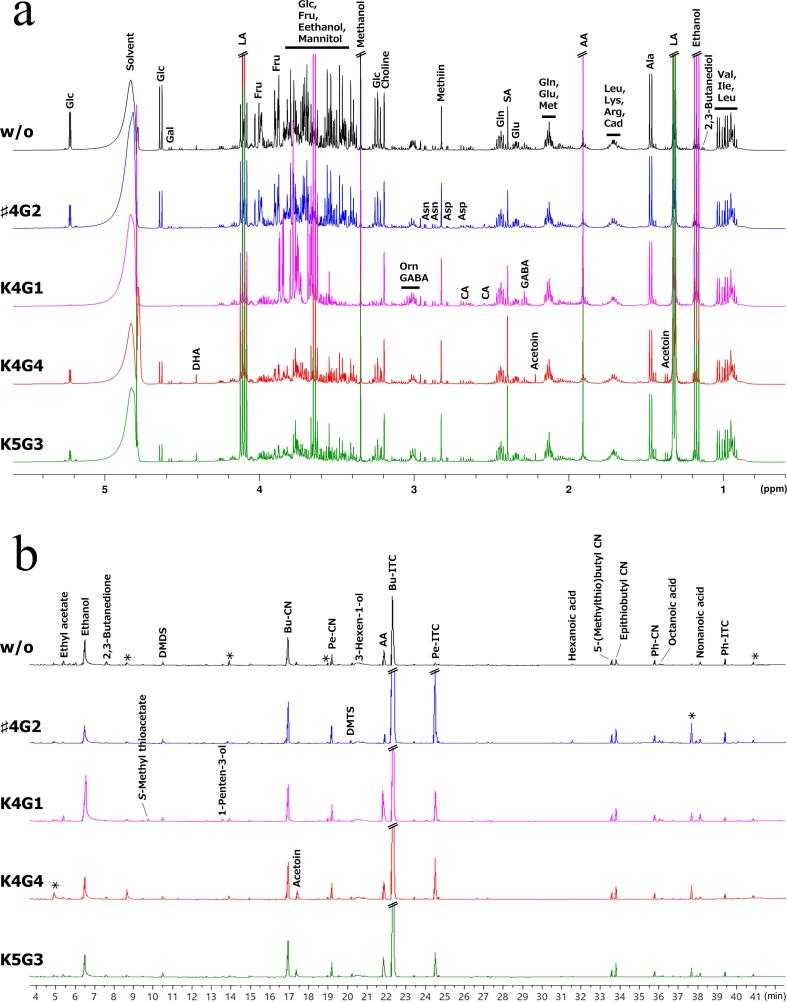
Compositional analyses of water-soluble and volatile compounds of *nozawana-zuke* pickling juice by NMR and SPME-GC/MS. (a) ^1^H NMR spectra and metabolite annotations of samples at 21 d. The spectral region ranging from 5.60 to 0.60 ppm is displayed. (b) Total ion chromatograms of SPME-GC/MS analysis and metabolite annotations. The labels U and asterisk (*) represent peaks of unannotated compounds and those observed in blank measurements, respectively. The following bacterial strains were used: ♯4G2, *Latilactobacillus curvatus*; K4G1, *Levilactobacillus brevis*; and K4G4 and K5G3, *Lactiplantibacillus plantarum*.

NMR and SPME-GC/MS were used to analyze the chemical compositions of water-soluble and volatile components, respectively. The dominant signals observed in the NMR spectra indicated that the major metabolites were organic acids: lactic acid (LA), acetic acid (AA), and succinic acid (SA); aldoses and alditols: glucose (Glc), fructose (Fru), and mannitol; alcohols: ethanol and methanol; amino acids: alanine (Ala), glutamic acid (Glu), glutamine (Gln), valine (Val), leucine (Leu), and isoleucine (Ile); and choline and methiin (Fig. 2a). Signals with lower intensities included citric acid (CA), formic acid, fumaric acid, γ-aminobutyric acid (GABA), galactose, trehalose, 2,3-butanediol, phenylalanine (Phe), tyrosine (Tyl), aspartic acid (Asp), asparagine (Asn), histidine (His), tryptophan (Trp), acetoin, ornithine (Orn), dihydroxyacetone (DHA), and uracil. In two-dimensional NMR spectra, more metabolites were annotated, including arabinose (Ara), *myo*-inositol, arginine (Arg), lysine (Lys), methionine (Met), glycine (Gly), serine (Ser), threonine (Thr), proline (Pro), pyroglutamic acid (Glp), and cadaverine (Cad) (Fig. S1).

The GC/MS chromatograms of the samples at 21 d showed that isothiocyanates (ITCs), including 3-butenyl ITC (Bu-ITC) and 4-pentenyl ITC (Pe-ITC), were the common major peaks, followed by AA, ethanol, and cyanides (CNs) such as 3-butenyl CN (Bu-CN) and 4-pentenyl CN (Pe-CN) (Fig. 2b). Lower-intensity peaks were observed for dimethyl disulfide (DMDS), dimethyl trisulfide (DMTS), 3-hexen-1-ol, phenethyl CN (Ph-CN), phenethyl ITC (Ph-ITC), 3,4-epithiobutyl-CN (epithiobutyl-CN), 5-(methylthio)butyl CN, octanoic acid, and nonanoic acid. The levels of ethyl acetate, 2,3-butanedione, *S*-methyl thioacetate, acetoin, and hexanoic acid varied among samples. The detected GC/MS peaks are listed in Table S1. Minute peaks were also observed, but these peaks did not contribute substantially to the differences among samples in the metabolomic analyses.

### 3.3 NMR metabolomics of fermented *nozawana-zuke*

**Fig. 3:**
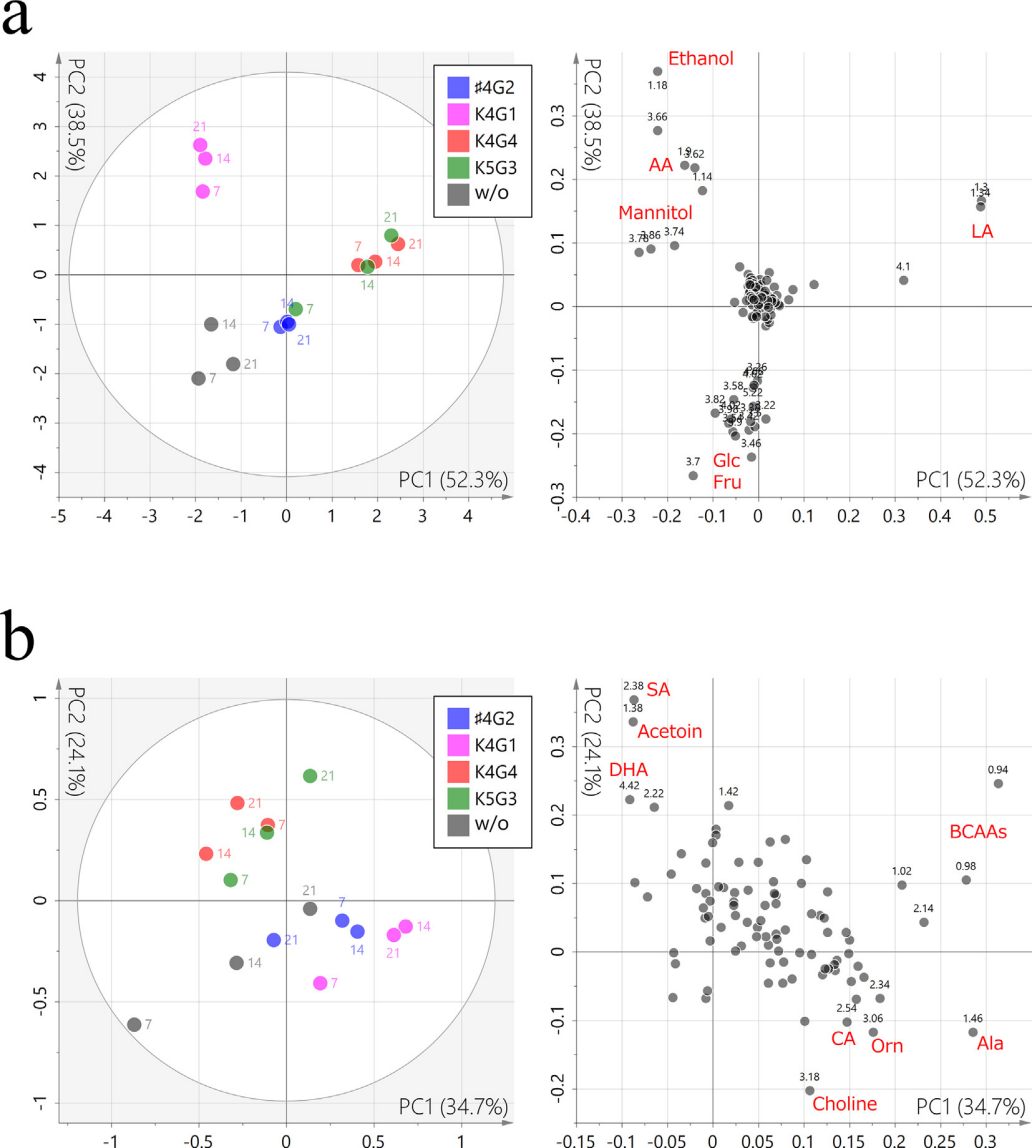
PCA of water-soluble compounds in *nozawana-zuke* samples. PC1–PC2 planes of score (left) and loading (right) plots obtained from (a) PCA and (b) two-step PCA. In the score plots, samples are color-coded as shown in the legend and labeled by fermentation duration in days. In the loading plots, numerical labels in black represent chemical shifts in ppm. The variables contributing to the feature space are labeled with metabolite names in red. The following bacterial strains were used: ♯4G2, *Latilactobacillus curvatus*; K4G1, *Levilactobacillus brevis*; and K4G4 and K5G3, *Lactiplantibacillus plantarum*. (For interpretation of the references to color in this figure legend, the reader is referred to the web version of this article.)

Differences in the chemical composition among the W/O and S/C samples were evaluated by PCA based on the dataset generated from the ^1^H NMR spectra. In a comparison applying all variables in the dataset (Fig. 3a), the first and second principal component (PC1 and PC2, 52.3% and 38.5% of the total variance, respectively) indicated that the differences depended more on the starter culture than on the fermentation duration. The loading plot explained the different contributions of the major signals observed in the NMR spectra. LA was strongly represented in *Lactiplantibacillus plantarum* K4G4 and K5G3 starter cultures, followed by that for *Latilactobacillus curvatus* ♯4G2. Ethanol, AA, and mannitol dominated in *Levilactobacillus brevis* K4G1 (Fig. 3a), whereas Glc and Fru dominated in W/O (Fig. 3a).

Two-step PCA was then conducted to assess the effects of variables presenting at lower intensities by excluding those derived from major components. The obtained feature space showed a class separation along the PC2 axis between *Lactiplantibacillus plantarum* K4G4 and K5G3 and *Latilactobacillus curvatus* ♯4G2, *Levilactobacillus brevis* K4G1, and W/O (Fig. 3b). SA, acetoin, and DHA contributed to the former class, whereas choline, Orn, GABA, and CA contributed to the latter class. PC1 accounted for differences within strains, such as fermentation duration, as indicated by Ala and branched-chain amino acids, including Val, Leu, and Ile.

### 3.4 SPME-GC/MS metabolomics of fermented *nozawana-zuke*

**Fig. 4:**
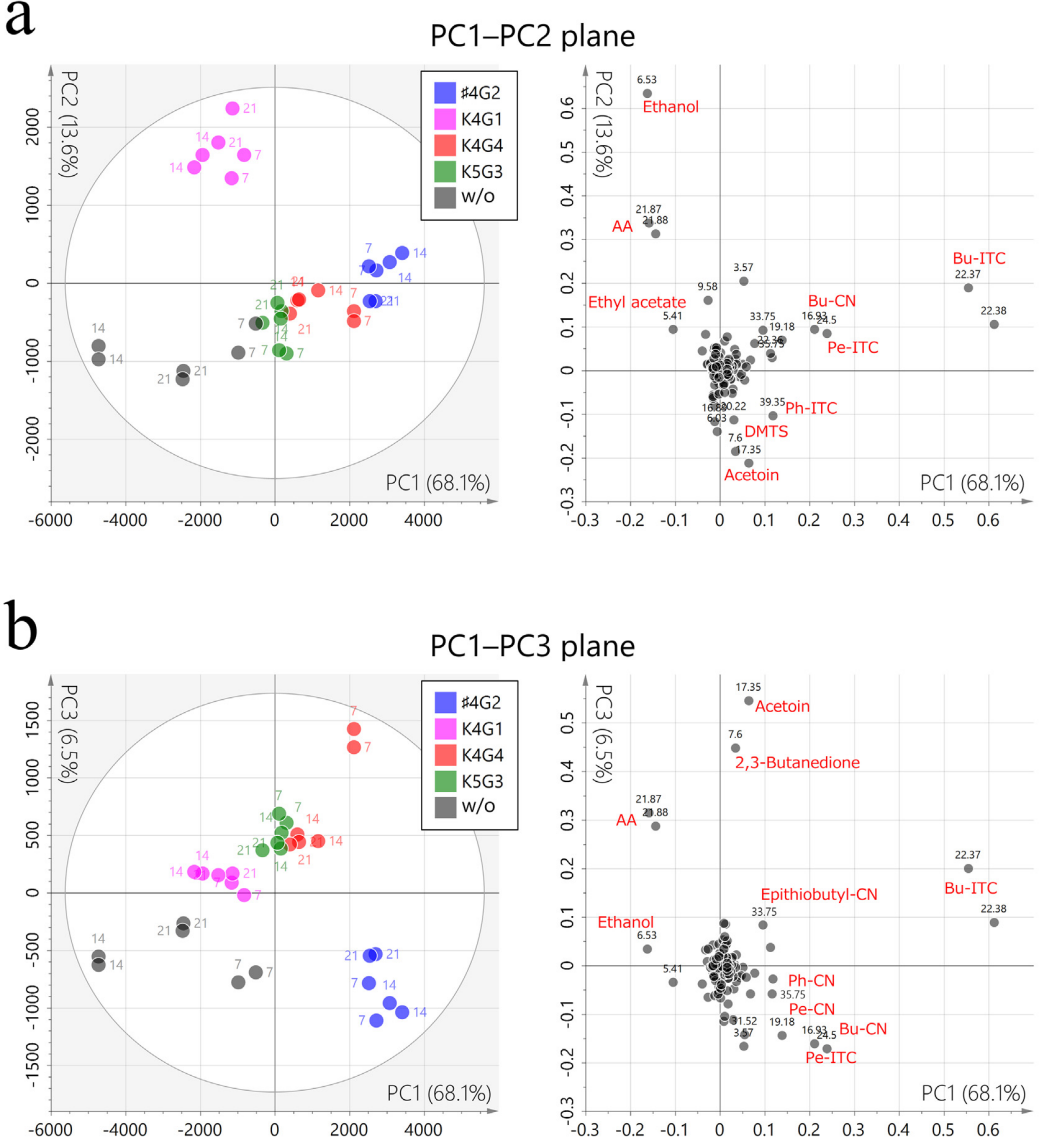
PCA of volatile compounds in *nozawana-zuke* samples. (a) PC1–PC2 and (b) PC1–PC3 planes of score (left) and loading (right) plots. In the score plots, the samples are color-coded as shown in the legend and labeled with fermentation duration in days. In the loading plots, numerical labels in black represent retention time in min. The variables contributing to the feature space are labeled with metabolite names in red. The following bacterial strains were used: ♯4G2, *Latilactobacillus curvatus*; K4G1, *Levilactobacillus brevis*; and K4G4 and K5G3, *Lactiplantibacillus plantarum*. (For interpretation of the references to color in this figure legend, the reader is referred to the web version of this article.)

We explored differences in GC/MS peak profiles among samples to examine the impact of fermentation with starter cultures versus that without starter cultures. In agreement with the PCA results of NMR metabolomics, GC/MS PCA data also revealed more pronounced differences by starter culture type rather than by fermentation duration. The PC1–PC2 plane of the score plot shows a sequential distribution of the strains *Lactiplantibacillus plantarum* K4G4 and K5G3 and *Latilactobacillus curvatus* ♯4G2 along PC1 and an isolated class of *Levilactobacillus brevis* K4G1 along PC2 (Fig. 4a). The loading plot indicated that ITCs and CNs contributed to the former, particularly to *Latilactobacillus curvatus* ♯4G2. *Levilactobacillus brevis* K4G1 was characterized by ethanol and AA, consistent with the NMR metabolomics findings. The samples of *Levilactobacillus brevis* K4G1 and W/O also showed the presence of ethyl acetate. Upon examination of the third principal component (PC3), a clear class separation of *Latilactobacillus curvatus* ♯4G2 from the others was observed (Fig. 4b). The samples were negatively characterized by low levels of acetoin and 2,3-butanedione, in addition to the presence of Pe-ITC and Bu-CN and Pe-CN.

### 3.5 Significance of metabolites and their time-course changes

**Fig. 5:**
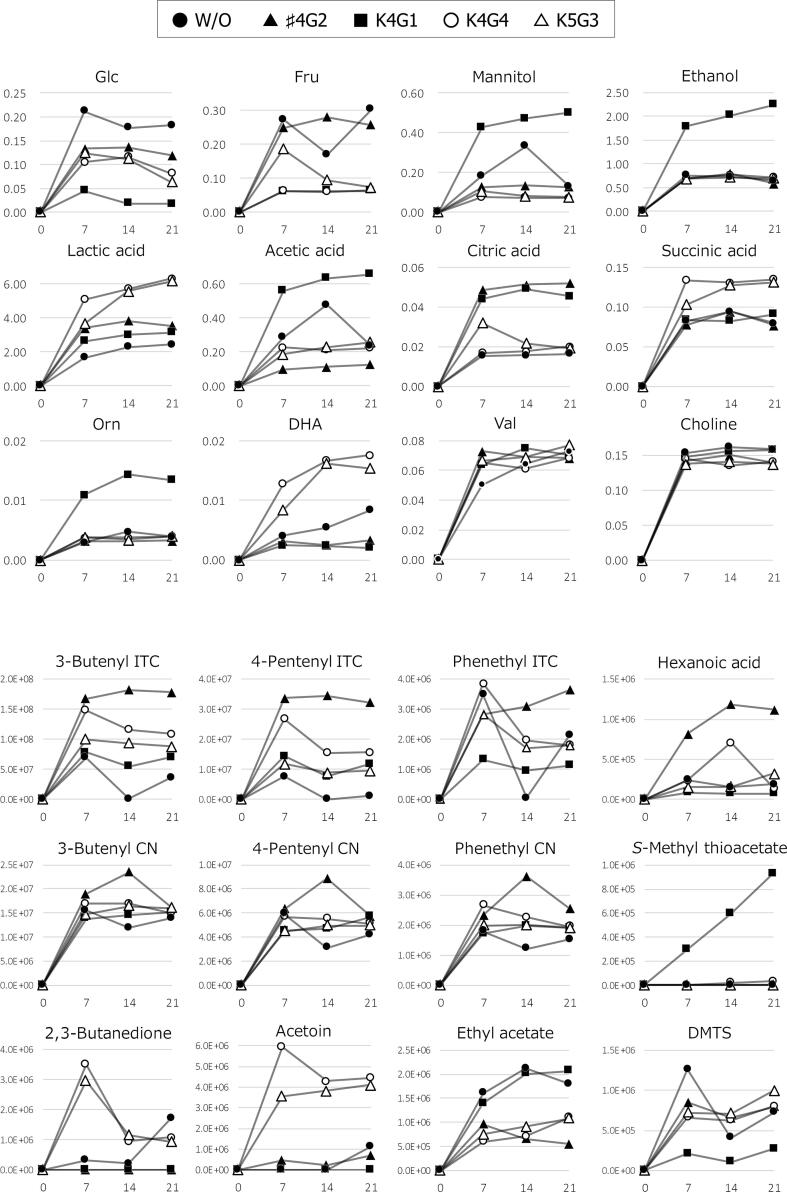
Changes to compounds in pickling juice during *nozawana-zuke* fermentation. The *x*-axis indicates fermentation duration in days, and the *y-*axis represents signal intensities of displayed metabolites. Symbols represent different bacterial strains, as shown in the legend. The initial addition of brine was considered day 0 and used as such to construct the plot.

The significance of the differences in the metabolites highlighted in the metabolomic analyses was assessed by subjecting the signal intensity calculated from the peaks of individual compounds to statistical analysis by multiple comparison tests (Table 1). Statistical significance was confirmed for most of the tested metabolites, but patterns of significance among groups were complex. Of these, differences separating the samples into more than three groups were significant for the levels of Glc, Fru, LA, AA, mannitol, choline, Bu-ITC, Pe-ITC, and ethyl acetate. We also found that certain metabolites were characteristic to certain strains, such as Bu-ITC, Pe-ITC, epithiobutyl-CN, and hexanoic acid for *Latilactobacillus curvatus* ♯4G2; AA, ethanol, mannitol, Orn, *S*-methyl thioacetate, and DMTS for *Levilactobacillus brevis* K4G1; and LA, SA, DHA, choline, acetoin, and 2,3-butanedione for *Lactiplantibacillus plantarum* strains K4G4 and K5G3. Conversely, the other metabolites highlighted by PCA, such as Val, Ile, Leu, Ala, Ph-ITC, Bu-CN, and Pe-CN, did not vary significantly among strains. Time-course data shown in Fig. 5 indicated that the levels of most metabolites in pickling juice, such as LA, AA, SA, ethanol, Orn, Bu-ICT, Bu-CN, hexanoic acid, and ethyl acetate, reached around maximum intensity by 7 d and remained at this intensity throughout the 3-week experimental period (Fig. 5). However, there were exceptions: the 2,3-butanedione intensity decreased sharply from 7 to 14 d in *Lactiplantibacillus plantarum* K4G4 and K5G3 and the *S*-methyl thioacetate intensity gradually increased over the 21-d period in *Levilactobacillus brevis* K4G1.

**Table 1:** Results of multiple comparison test for the compounds highlighted in metabolomic characterization.

	Signal intensity ± standard deviation*				
Compound	W/O starter culture	*Latilactobacillus curvatus* ♯4G2	*Levilactobacillus brevis* K4G1	*Lactiplantibacillus plantarum* K4G4	*Lactiplantibacillus plantarum* K5G3
NMR peaks					
Glc	0.190 ± 0.019 ^a^	0.130 ± 0.009 ^b^	0.027 ± 0.016 ^c^	0.101 ± 0.018 ^b^	0.100 ± 0.032 ^b^
Fru	0.248 ± 0.069 ^a^	0.262 ± 0.016 ^b^	0.062 ± 0.001 ^c^	0.063 ± 0.003 ^c^	0.117 ± 0.060 ^c^
LA	2.131 ± 0.406 ^a^	3.596 ± 0.223 ^ab^	2.928 ± 0.272 ^a^	5.706 ± 0.606 ^c^	5.126 ± 1.311 ^bc^
AA	0.335 ± 0.127 ^a^	0.113 ± 0.013 ^bc^	0.617 ± 0.050 ^d^	0.221 ± 0.007 ^ac^	0.225 ± 0.036 ^ac^
CA	0.016 ± 0.001 ^a^	0.051 ± 0.002 ^b^	0.046 ± 0.003 ^b^	0.018 ± 0.002 ^a^	0.024 ± 0.007 ^a^
SA	0.085 ± 0.008 ^a^	0.083 ± 0.010 ^a^	0.086 ± 0.005 ^a^	0.133 ± 0.002 ^b^	0.121 ± 0.015 ^b^
Ethanol	0.712 ± 0.059 ^a^	0.689 ± 0.107 ^a^	2.016 ± 0.230 ^b^	0.728 ± 0.038 ^a^	0.699 ± 0.015 ^a^
Mannitol	0.216 ± 0.105 ^a^	0.129 ± 0.004 ^ab^	0.468 ± 0.037 ^c^	0.073 ± 0.002 ^bd^	0.087 ± 0.015 ^ad^
Orn	nd	nd	0.013 ± 0.002	nd	nd
DHA	0.006 ± 0.002 ^a^	nd	nd	0.016 ± 0.003 ^b^	0.013 ± 0.004 ^b^
Ala	0.286 ± 0.034 ^ab^	0.326 ± 0.018 ^a^	0.297 ± 0.009 ^ab^	0.261 ± 0.016 ^b^	0.281 ± 0.014 ^ab^
Val	0.062 ± 0.011	0.070 ± 0.002	0.070 ± 0.006	0.065 ± 0.004	0.071 ± 0.006
Ile	0.049 ± 0.009	0.056 ± 0.003	0.053 ± 0.004	0.062 ± 0.005	0.062 ± 0.004
Leu	0.190 ± 0.043	0.233 ± 0.010	0.233 ± 0.028	0.229 ± 0.016	0.242 ± 0.023
Choline	0.157 ± 0.004 ^a^	0.144 ± 0.006 ^bc^	0.153 ± 0.006 ^ab^	0.140 ± 0.005 ^c^	0.138 ± 0.002 ^c^

GC/MS peaks^**^					
Bu-ITC	35659 ± 34193 ^a^	175589 ± 7433 ^b^	67603 ± 12035 ^ac^	124327 ± 21067 ^bd^	93700 ± 5806 ^cd^
Pe-ITC	2984 ± 4070 ^a^	33411 ± 1103 ^b^	11211 ± 3442 ^ac^	19263 ± 6457 ^c^	10121 ± 1550 ^ac^
Ph-ITC	2438 ± 927 ^ab^	3182 ± 409 ^a^	1140 ± 185 ^b^	2538 ± 1136 ^ab^	2103 ± 618 ^ab^
Bu-CN	13742 ± 1819 ^a^	19531 ± 3678 ^b^	14464 ± 686 ^ab^	16284 ± 1124 ^ab^	15684 ± 912 ^ab^
Pe-CN	4399 ± 1420	6963 ± 1668	4966 ± 622	5432 ± 290	4791 ± 288
Ph-CN	1509 ± 290 ^a^	2825 ± 685 ^b^	1859 ± 130 ^ab^	2289 ± 350 ^ab^	1960 ± 49 ^ab^
Epithiobutyl-CN	2474 ± 241 ^a^	5964 ± 394 ^b^	4176 ± 177 ^a^	5374 ± 1058 ^b^	4311 ± 1530 ^a^
Acetoin	376 ± 651 ^a^	471 ± 240 ^a^	nd	4902 ± 919 ^b^	3832 ± 272 ^b^
2,3-Butanedione	756 ± 843	nd	nd	1852 ± 1439	1691 ± 1104
Ethyl acetate	1838 ± 262 ^a^	723 ± 216 ^b^	1820 ± 370 ^a^	804 ± 263 ^bc^	912 ± 168 ^bc^
Hexanoic acid	195 ± 47 ^a^	1039 ± 197 ^b^	79 ± 6 ^a^	363 ± 304 ^a^	212 ± 97 ^a^
*S*-Methyl thioacetate	nd	nd	606 ± 314 ^a^	20 ± 18 ^b^	nd
DMTS	796 ± 431 ^a^	765 ± 100 ^a^	194 ± 80 ^b^	696 ± 93 ^a^	807 ± 159 ^a^

*The average intensities of the three samples from each strain. The superscript letters indicate significant differences (*p* < 0.05) by Tukey's multiple comparison test.

^**^The intensity values of GC/MS peaks are displayed in units of 1000. nd = not detected.

## 4 Discussion

Overall, the results indicated that starter cultures were associated with better fermentation progress, stable cell counts, and compositional profile variation in *nozawana-zuke* samples. The findings strongly suggested that the application of starter culture LAB improved the quality of fermented *nozawana-zuke*. To the best of our knowledge, this is the first study to assess Japanese salted pickle made from *Brassica rapa* L., including *nozawana-zuke*, via a metabolomics approach. The variation in the compositional profile could also be associated with differences in sensory quality. The overview of the comprehensive profiles of water-soluble and volatile compounds in this study may help in understanding the potential starter culture-dependent effects on the chemical characteristics and resulting quality of fermented pickle products.

In contrast to the S/C *nozawana-zuke* samples, the W/O samples exhibited delayed fermentation, higher terminal pH, more residual sugars, and lower overall acid production. The W/O samples also did not maintain viable LAB counts after the first 7 d of the 3-week fermentation period. These findings corroborate those observed in the starter culture fermentation of Chinese sauerkraut (Xiong, Li, Guan, Peng, & Xie, 2014), suggesting that autochthonous LAB with acid susceptibility, such as heterolactic-fermentative *Leuconostoc mesenteroides*, only grew transiently. The results of metabolomics analyses indicated that the chemical profile in W/O samples clearly differed to that in S/C samples. In terms of volatile compounds, W/O samples were characterized by substantially lower levels of Bu-ITC, Pe-ITC, and epithiobutyl-CN. Relative to those in S/C samples, the ratios of Bu-ITC, Pe-ITC, and epithiobutyl-CN levels in W/O samples were only 0.20–0.53-fold, 0.09–0.29-fold, and 0.41–0.59-fold, respectively. ITCs and CNs are major aroma-active volatiles in cruciferous vegetable pickles and are degraded from glucosinolates by the catalytic action of myrosinase (β-thioglucosidase glucohydrolase; EC 3.2.3.1) (Latte, Appel, & Lampen, 2011). The intensity of ethyl acetate, a well-known aroma-active component in fermented pickles (Yang et al., 2020, Zhao et al., 2016), was 2.02–2.54-fold higher in the W/O sample than in the S/C samples, except for *Levilactobacillus brevis* K4G1. This distinct profile with these major aroma-active volatiles may result in odor differences between the S/C and W/O samples. In fact, after the fermentation of W/O samples, the samples were found to emit an unpleasant smell accompanied by a strong fermentation odor. As a potential off-flavor component in *nozawana-zuke*, epithiobutyl-CN has been identified to negatively contribute to the flavor via its heavy, stale odor (Uda, Suzuki, & Maeda, 1992). However, the level of this volatile was lower in W/O samples than in S/C samples in this study. This might suggest the possibility that the fermentation odor in W/O samples is influenced by other volatile compound(s) derived from starter culture fermentation.

Fermentation with *Latilactobacillus curvatus* ♯4G2, a facultative heterofermentative species, resulted in moderate titration acidity (Fig. 1), which may contribute to a mild sour taste. Fermentation with this strain was associated with higher levels of residual sugars (particularly Fru) that persisted through the 21-d fermentation period. Because *Latilactobacillus curvatus* can produce acid from Fru but is relatively sensitive to low pH (Klein, Dicks, Pack, Hack, Zimmermann, Dellaglio, & Reuter, 1996), the moderate LA production from Glc may have inhibited the cells and its Fru metabolization during fermentation, resulting in lower acid accumulation than that in *Lactiplantibacillus plantarum* strains K4G4 and K5G3. In addition to the lower acidity, *Latilactobacillus curvatus* ♯4G2 exhibited a characteristic volatile profile. The level of AA, a key flavor compound that confers a sour pickle-like smell (Zhao, et al., 2016), was the lowest in the ♯4G2 sample, potentially because of the homolactic fermentation pathway of this species. In *Latilactobacillus curvatus* ♯4G2, higher levels of ITCs and lower intensities of acetoin and 2,3-butanedione also contributed to the characteristic volatile profile. The levels of Bu-ITC, Pe-ITC, and Ph-ITC in the ♯4G2 sample were higher than those in the other S/C samples by 1.41–2.60-fold, 1.73–3.30-fold, and 1.25–2.79-fold, respectively. Tolonen et al. reported similar observations for sauerkraut fermentation, obtaining maximal levels of ITCs upon fermentation with *Latilactobacillus sakei* (Tolonen et al., 2004), a species closely related to *Latilactobacillus curvatus* (Klein, et al., 1996). The degradation products of glucosinolate strongly depend on its side chain properties and reaction conditions, including pH value. ITC primarily forms at pH 6–7, whereas CN is favored at pH 2–5 (Latte et al., 2011). The levels of ITCs detected in this study, however, did not directly correlate with the pH values, implying that ITC levels were influenced depending on the starter culture. Although ITCs are known to be degradation products of glucosinolate derived from the enzymic reaction in raw material, it is intriguing that certain bacterial species can possibly influence ITC levels in fermented pickles. In addition, *Latilactobacillus curvatus* ♯4G2 might engage a bacterial myrosinase in *nozawana-zuke* fermentation as glucosinolate degradation has been reported in several *Lactobacillus* spp. (Nugon-Baudon et al., 1990, Palop et al., 1995), although it has not been reported for *Latilactobacillus curvatus*. The persistent CA content during fermentation with *Latilactobacillus curvatus* ♯4G2 indicates that this strain lacks CA assimilation ability in *nozawana-zuke* fermentation. This result may be associated with the fact that the levels of acetoin and 2,3-butanedione were low because these compounds are conversion products from CA (Gänzle, 2015).

In contrast to samples fermented with *Latilactobacillus curvatus* ♯4G2, samples fermented with *Lactiplantibacillus plantarum* strains K4G4 and K5G3 showed CA catabolism, resulting in high levels of the conversion products, acetoin and 2,3-butanedione (also known as diacetyl). Acetoin and 2,3-butanedione are strong aroma-active volatiles and are associated with yogurt and butter-like odors (Gänzle, 2015). These are desirable in fermented dairy foods and wine but not in beer and Japanese sake. Although these volatiles are commonly found in fermented vegetable pickles, their influence on the flavor quality of *nozawana-zuke* remains unclear. The vigorous fermentation of Glc and Fru and the strong resistance to acids led to an abundance of LA and a particularly low terminal pH value. Excess lactic fermentation is generally not preferred in fermented pickles. Their strong pH-lowering capacity, however, could be useful in *nozawana-zuke* production to increase preservability during storage and aid fermentation in difficult conditions, such as conditions with limited amounts of fermentable sugars and nutrients. Samples fermented with both *Lactiplantibacillus plantarum* strains were also characterized by increased production of DHA, as reported in a study of fermented mixed vegetables (Kim, Choi, Park, & Kim, 2019). DHA is associated with a sweet, cooling taste and ether-type odor and is known to affect the sensory quality of wine (Du Toit & Pretorius, 2000); however, its effect on fermented pickle taste remains unclear.

The sample fermented with *Levilactobacillus brevis* K4G1 presented with a metabolite profile that was different from that of samples fermented with the other strains. *Levilactobacillus brevis* is an obligatory heterofermentative species that ferments Glc and produces LA together with AA, ethanol, and CO_2_. In the *nozawana-zuke* fermentation in this study, heterolactic fermentation may have caused moderate pH reduction and acidity, contrary to the highest consumption of Glc and Fru among the four strains. Fru was likely not directly utilized for lactic fermentation but instead for mannitol interconversion to maintain the bacterial redox balance (Gänzle, 2015). The intensities of ITCs were the lowest among the S/C samples (Fig. 5, Table 1), whereas that of ethyl acetate, a fruity aroma metabolite, was substantially higher than that in the samples fermented with other strains (at 2.06–2.59-fold). With respect to other compounds, it is noteworthy that fermentation with *Levilactobacillus brevis* K4G1 influences the levels of DMTS and *S*-methyl thioacetate. *S*-methyl thioacetate has been detected in Chinese fermented pickle (paocai) and cheese (Peres et al., 2001, Zhao et al., 2016). The present study, however, is the first to report that fermentation with *Levilactobacillus brevis* specifically resulted in *S*-methyl thioacetate accumulation in pickles. Sulfur compounds are associated with unpleasant odors at a very low sensory threshold (Zhao, et al., 2016). The control of sulfur compound composition by starter culture LAB could be useful for flavor modification of fermented pickles. In cabbage sauerkraut, sulfur-containing volatiles such as DMDS and DMTS are derived from *S*-methyl-l-cysteine sulfoxide in raw material through mechanical damage and heating without bacterial reaction (Chin & Lindsay, 1994). Further investigation is necessary to assess the starter culture-dependent influence on the changes in sulfur-containing volatile composition in *nozawana-zuke* fermentation.

In conclusion, this study assessed the impact of various starter culture LAB on water-soluble and volatile compound profiles of fermented *nozawana-zuke*, compared these profiles with those obtained after fermentation without a starter culture, and further evaluated these results using a metabolomics approach. Our findings demonstrate that the levels of a wide range of metabolites in *nozawana-zuke* could be controlled using a specific starter culture strain. Many of the metabolites highlighted herein are taste- and/or aroma-active compounds, which suggests the possibility that the selection of starter culture can strongly influence the sensory profile of fermented *nozawana-zuke* products. Specifically, our results suggest that starter-culture-dependent changes in glucosinolate degradation products should be assessed in greater depth to examine their contributions to taste and aroma. In addition, we observed that different starter cultures produce different levels of beneficial components, such as Orn and GABA, indicating the starter culture-dependent influence on health benefits of fermented *nozawana-zuke* in addition to immunomodulatory effects reported in previous studies (Kawahara and Otani, 2006, Sandagdorj et al., 2019). Further research is needed to substantiate the preliminary findings obtained in this study regarding the impact on flavor and health benefits. A wide range of LAB are used in the fermentation of vegetable pickles, and their fermentation characteristics differ even among strains of the same species. High-throughput screening by metabolomic analysis could facilitate a broader choice of starter culture candidates and contribute to improving the quality of fermented pickles.

## Declaration of Competing Interest

The authors declare that they have no known competing financial interests or personal relationships that could have appeared to influence the work reported in this paper.

## Appendix A Supplementary data

Supplementary data 1 ^1^H–^13^C HSQC spectrum and metabolite annotation of nozawana-zuke pickling juice. Data represent the results for the sample fermented without a starter culture (W/O) over 21 d.

Supplementary data 2 Annotated volatile compounds in nozawana-zuke pickling juice.
