# Supplementary figures and images for: Metabolomic evaluation of different starter culture effects on water-soluble and volatile compound profiles in nozawana pickle fermentation
Source: Food Chem (Oxf). 2021 Mar 17;2:100019. doi: 10.1016/j.fochms.2021.100019 (PMC8991705; doi:10.1016/j.fochms.2021.100019)

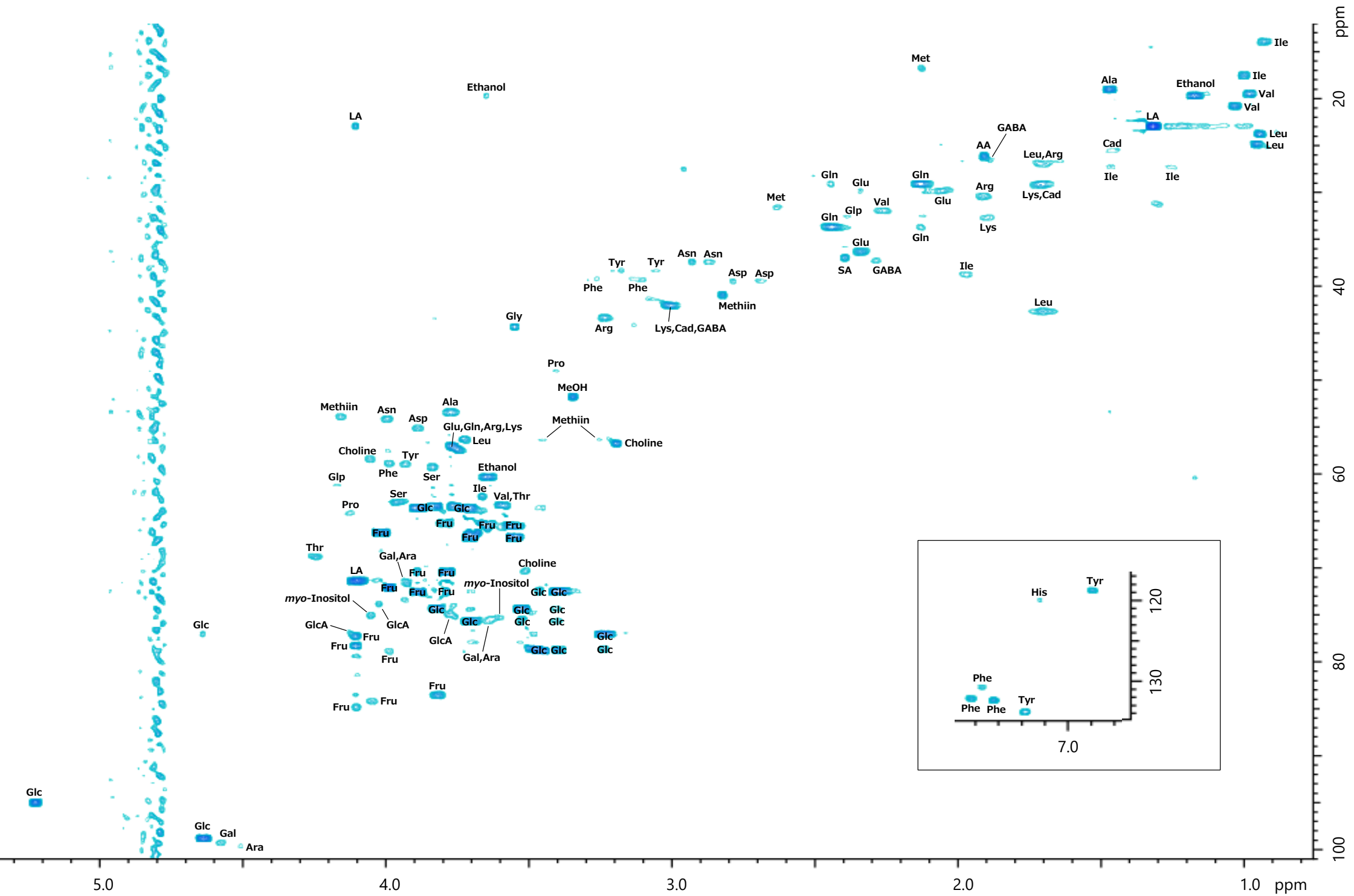

Supplement: Supplementary data 1 — 1H–13C HSQC spectrum and metabolite annotation of nozawana-zuke pickling juice. Data represent the results for the sample fermented without a starter culture (W/O) over 21 d. [file mmc1.pdf]
