# Supplementary material for: Metabolomic evaluation of different starter culture effects on water-soluble and volatile compound profiles in nozawana pickle fermentation
Source: Food Chem (Oxf). 2021 Mar 17;2:100019. doi: 10.1016/j.fochms.2021.100019 (PMC8991705; doi:10.1016/j.fochms.2021.100019)
Supplement: Supplementary data 2 — Annotated volatile compounds in nozawana-zuke pickling juice. [file mmc2.docx]

**Table S1. Annotated volatile compounds in *nozawana-zuke* pickling juice**

|  |  |  | Result of MS similarity search | |
| --- | --- | --- | --- | --- |
| Peak # | RT (min) | RI | Similarity index | Annotation^a^ |
| 1 | 3.57 | 764 | 96 | Carbon disulfide |
| 2 | 4.27 | 808 | 96 | Acetone |
| 3 | 4.45 | 819 | 97 | Methyl acetate |
| 4 | 5.02 | 855 | - | U |
| 5 | 5.41 | 879 | 96 | Ethyl acetate |
| 6 | 5.63 | 893 | 94 | 2-Butanone |
| 7 | 5.74 | 899 | 97 | 2-Methyl-2-propanol |
| 8 | 5.93 | 907 | 97 | 2-Methylbutanal |
| 9 | 6.03 | 910 | 95 | 3-Methylbutanal |
| 10 | 6.35 | 922 | 96 | Isopropyl alcohol |
| 11 | 6.52 | 929 | 97 | Ethanol |
| 12 | 7.61 | 969 | 97 | 2,3-Butanedione |
| 13 | 8.46 | 1000 | 94 | Methyl isobutyl ketone |
| 14 | 8.71 | 1008 | 96 | Amylene hydrate |
| 15 | 9.76 | 1040 | 93 | *S*-Methyl thioacetate |
| 16 | 10.51 | 1062 | 93 | Dimethyl disulfide |
| 17 | 10.56 | 1064 | - | U |
| 18 | 10.83 | 1072 | 86 | Hexanal |
| 19 | 13.57 | 1155 | 98 | 1-Penten-3-ol |
| 20 | 14.15 | 1173 | 95 | 2-Heptanone |
| 21 | 14.91 | 1196 | 94 | 4-Methyl-2-heptanone |
| 22 | 16.78 | 1256 | 87 | Butyl isothiocyanate |
| 23 | 16.94 | 1261 | 96 | 3-Butenyl cyanide |
| 24 | 17.35 | 1274 | 98 | Acetoin |
| 25 | 17.76 | 1288 | 94 | Pentyl cyanide |
| 26 | 14.95 | 1197 | - | U |
| 27 | 19.18 | 1336 | 94 | 4-Pentenyl cyanide |
| 28 | 19.53 | 1348 | 93 | 4-Methylpentyl cyanide |
| 29 | 19.64 | 1352 | - | U |
| 30 | 20.22 | 1372 | 96 | Dimethyl trisulfide |
| 31 | 20.48 | 1381 | 95 | 3-Hexen-1-ol |
| 32 | 20.56 | 1384 | 95 | Nonanal |
| 33 | 20.82 | 1392 | 94 | Hexyl cyanide |
| 34 | 21.85 | 1430 | 98 | Acetic acid |
| 35 | 22.33 | 1450 | 92 | 3-Butenyl isothiocyanate |
| 36 | 22.65 | 1459 | - | U |
| 37 | 23.95 | 1508 | - | U |
| 38 | 24.05 | 1512 | 97 | Benzaldehyde |
| 39 | 24.48 | 1529 | 0.86^b^ | 4-Pentenyl isothiocyanate |
| 40 | 24.67 | 1537 | 97 | Linalol |
| 41 | 26.64 | 1615 | - | U |
| 42 | 27.20 | 1638 | 97 | Tolualdehyde |
| 43 | 31.52 | 1827 | 97 | Hexanoic acid |
| 44 | 33.55 | 1919 | 82 | 5-(Methylthio)butyl cyanide^c,e,f^ |
| 45 | 33.76 | 1930 | - | 3,4-Epithiobutyl cyanide^d,e,f^ |
| 46 | 35.74 | 2025 | 97 | Phenethyl cyanide |
| 47 | 36.00 | 2036 | 97 | Octanoic acid |
| 48 | 36.14 | 2044 | - | U |
| 49 | 36.68 | 2069 | - | U |
| 50 | 37.88 | 2127 | - | U |
| 51 | 38.06 | 2135 | 94 | Nonanoic acid |
| 52 | 39.36 | 2197 | 97 | Phenethyl isothiocyanate |
| 53 | 40.02 | 2229 | - | U |
| 54 | 40.65 | 2260 | - | U |
| 55 | 40.79 | 2266 | - | U |

^a^ U: Unannotated by mass spectrum and retention index.

^b^ Similarity score calculated by MassBank spectrum search (<https://massbank.eu/MassBank/>).

^c^ Iranshahi, M. (2012). A review of volatile sulfur-containing compounds from terrestrial plants: biosynthesis, distribution and analytical methods. *Journal of Essential Oil Research*, 24(4), 393-434.

^d^ Kato, M., Imayoshi, Y., Iwabuchi, H., & Shimomura, K. (2011). Kinetic changes in glucosinolate-derived volatiles by heat-treatment and myrosinase activity in nakajimana (*Brassica rapa* L. cv. *nakajimana*). *Agricultural and Food Chemistry*, 59, 11034-11039.

^e^ Spencer, G. F., & Daxenbichler, M. E. (1980). Gas chromatography-mass spectrometry of nitriles, isothiocyanates and oxazolidinethiones derived from Cruciferous glucosinolates. *Journal of the Science of Food and Agriculture*, 31(4), 359-367.

f Uda, Y., Yoshida, S., Goto, M., & Egashira, H. (2007). Glucosinolate-derived volatiles in raw vegetables, fujisawakabu and yukina, and their pickled or salted products. *Nippon Shokuhin Kagaku Kogaku Kaishi*, 53(12), 559-562.
